# Altered Brain Activity and Effective Connectivity within the Nonsensory Cortex during Stimulation of a Latent Myofascial Trigger Point

**DOI:** 10.1155/2022/4416672

**Published:** 2022-08-12

**Authors:** Xinglou Li, Meiling Luo, Yan Gong, Ning Xu, Congcong Huo, Hui Xie, Shouwei Yue, Zengyong Li, Yonghui Wang

**Affiliations:** ^1^Rehabilitation Center, Qilu Hospital of Shandong University, Jinan, Shandong 250012, China; ^2^Department of Physical Medicine and Rehabilitation, The Affiliated Suzhou Hospital of Nanjing Medical University, Suzhou, Jiangsu 215000, China; ^3^School of Rehabilitation Medicine of Shandong University of Traditional Chinese Medicine, Jinan, Shandong 250355, China; ^4^Key Laboratory for Biomechanics and Mechanobiology of Ministry of Education, School of Biological Science and Medical Engineering, Beihang University, Beijing 100086, China; ^5^Beijing Key Laboratory of Rehabilitation Technical Aids for Old–Age Disability, National Research Center for Rehabilitation Technical Aids, Beijing 100176, China; ^6^Key Laboratory of Neuro-functional Information and Rehabilitation Engineering of the Ministry of Civil Affairs, Beijing 100176, China

## Abstract

Myofascial trigger point (MTrP), an iconic characteristic of myofascial pain syndrome (MPS), can induce cerebral cortex changes including altered cortical excitability and connectivity. The corresponding characteristically reactive cortex is still ambiguous. Seventeen participants with latent MTrPs underwent functional near-infrared spectroscopy (fNIRS) to collect cerebral oxygenation hemoglobin (*Δ*[oxy-Hb]) signals. The *Δ*[oxy-Hb] signals of the left/right prefrontal cortex (L/R PFC), left/right motor cortex (L/R MC), and left/right occipital lobe (L/R OL) of the subjects were measured using functional near-infrared spectroscopy (fNIRS) in the resting state, nonmyofascial trigger point (NMTrP), state and MTrP state. The data investigated the latent MTrP-induced changes in brain activity and effective connectivity (EC) within the nonsensory cortex. The parameter wavelet amplitude (WA) was used to describe cortical activation, EC to show brain network connectivity, and main coupling direction (mCD) to exhibit the dominant connectivity direction in different frequency bands. An increasing trend of WA and a decreasing trend of EC values were observed in the PFC. The interregional mCD was primarily shifted from a unidirectional to bidirectional connection, especially from PFC to MC or OL, when responding to manual stimulation during the MTrP state compared with resting state and NMTrP state in the intervals III, IV, and V. This study demonstrates that the nonsensory cortex PFC, MC, and OL can participate in the cortical reactions induced by stimulation of a latent MTrP. Additionally, the PFC shows nonnegligible higher activation and weakened regulation than other brain regions. Thus, the PFC may be responsible for the central cortical regulation of a latent MTrP. This trial is registered with ChiCTR2100048433.

## 1. Introduction

Myofascial trigger points (MTrPs) are limited sensitive points and can be found within almost any strained muscle, thus leading to the most extensive neck, shoulder, waist, and leg pain [1]. As the main obstacles to a better myofascial pain syndrome (MPS) clinical outcome [2], MTrPs will induce local pain, local convulsive response, and autonomic nerve phenomena once provoked and can be active and latent. Latent MTrPs commonly exist in healthy people and patients with musculoskeletal pain, leading to sensory-motor dysfunction. Compared with the active MTrPs, latent MTrPs can also play an essential role in characteristic neuromuscular excitability [3]. Mechanisms involved in MTrP production, including the “integrated hypothesis” and “energy crisis theory,” have been generally accepted [4, 5]. After cortical activation, central sensitization also contributes to the emergence of MTrPs [6]. Treatments focusing on the local MTrP, such as dry needling, shock wave therapy, and local block therapy, are the most common strategies [7]. However, there is a lack of specific and effective treatment, with pain easily recurring.

MTrPs can induce a complex multidimensional emotional experience, which relies on the neuronal activity of multiple regions of the cerebral cortex to complete the process of sensory, emotional, and cognitive responses [8]. However, there is no specific central nervous system for pain or nociception within the brain, and the cortical activation patterns can vary with different clinical pain symptoms. Although the somatosensory cortex can reflex pain signals, it is mainly responsible for perceiving pain, especially for identifying the location of pain in the body [9]. The complicated cortical pain processing needs further investigation as specific pain biomarkers and target treatments are lacking.

In recent years, the nonsensory cortex, particularly the prefrontal cortex (PFC), motor cortex (MC), and occipital lobe (OL), has been reported to play increasingly indispensable roles in pain processing. A series of brain imaging studies demonstrated that the PFC participates in neuropathic and musculoskeletal pain production. The PFC is also where pain information (painful and nociceptive stimuli) and others (including memory, emotion, and space) are integrated and processed [10]. Excitability changes in the MC are related to the severity of pain intensity, hyperalgesia, and allodynia [11]. Additionally, activation of the MC can also have an analgesic effect on chronic pain [12]. Thus, the MC has been a common target area for pain research, due to its connection with the nociceptive system and the effect of pain on motor function. Although the OL is not a typical “pain matrix” or salient network member, it can also be involved in the central processing of pain signals. The excitability of the OL, demonstrated by the electrophysiological images in patients with migraine and visual snow syndrome, has proved the loss of pain habituation and a lower pain threshold [13]. One study of 114 subjects has further illustrated that thermal pain stimuli can activate the pain matrix, while OL neuronal activity was observed inhibited [14]. Still, however, the cortical changes in the nonsensory cortical associated with the latent MTrPs are not understood.

Several brain imaging techniques, such as functional magnetic resonance imaging (fMRI) [15], positron emission tomography (PET) [16], and magnetoencephalography (MEG) [17], are the most available devices to collect pain-related cortical neuronal activity data. However, they are not suitable for the clinical rapid and patient-centric brain assessment. In this decade, functional near-infrared spectroscopy (fNIRS), supported by portability, affordability, and resistance to motion artifacts, has been suggested to assess neuronal activity by recording oxygenated and deoxygenated hemoglobin changes in cerebral tissues. As a noninvasive optical imaging technique, fNIRS can be applied in the clinic to monitor the human brain function changes [18]. This study was aimed at investigating the functionality of fNIRS to explore pain-related cortical activity.

The nonsensory cortex can elicit a large amount of information correlated to its role in the pain process. However, this informative resource has not been characterized in MTrP-related pain. This study stimulates the MTrP and NMTrP of recruited participants and was aimed at exploring the latent MTrP-induced changes of activation and network connections within the nonsensory cerebral cortex by an fNIRS.

## 2. Materials and Methods

### 2.1. Participants

This study is prospective and observational, involving a single-center, self-control, and single-blinding clinical trial. This project uses right brachioradialis, usually more frequently used and susceptible to lateral epicondylalgia than the left one, to study latent MTrPs [19].

The subjects are screened according to the following inclusion criteria: (1) no mental health diagnoses have been founded; (2) no medicine has been taken this 48 h; (3) age ranged from 20 to 50 years for both genders; (4) minimum primary school education; and (5) a latent MTrP is on the right brachioradialis but absent from the left side (there is no pain sensation in daily life, but pain could be stimulated by pressing or other methods) [20, 21]. The exclusion criteria are as follows: (1) pain resulting from other causes (such as rheumatic diseases, malignant tumors, and other infections); (2) disoriented subjects; (3) and subjects with more severe health comorbidities (such as heart, liver, lung, kidney, and other organs dysfunction). All participants signed a written informed consent before inclusion.

From July to October 2021, 17 participants were identified according to our study protocol. The study cohort consisted of 15 males and two females, with an average age of 25.41 ± 5.14 years. Other fundamental parameter indexes, such as visual analogue scale (VAS), blood pressure (BP), and heart rate (HR), also had been recorded when initially enrolled.

### 2.2. Experimental Design

**Figure 1:**
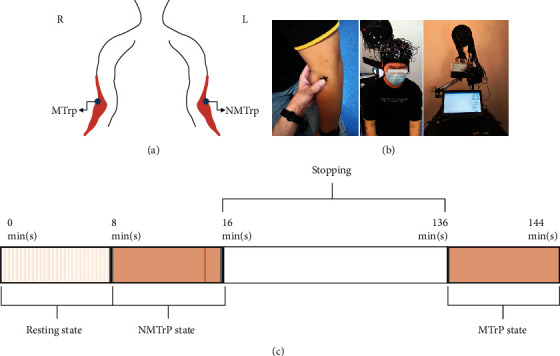
Schematic diagram of the experimental setup. (a) The distribution of MTrP and NMTrP: the marked blue regions of the bilateral brachioradialis muscle are the corresponding MTrP and NMTrP. (b) Experimental illustration. (c) Experimental schedule. NMTrP: nonmyofascial trigger point; MTrP: myofascial trigger point. Abbreviations: min(s), minute(s).

Firstly, the examiner pressed the right brachioradialis with bare hands to determine the position of the potential MTrP and then marked it with a marker pen. The latent MTrP could be diagnosed manually if these criteria were present: taut band, hypersensitive spot, and local twitch response during pressing. Symmetrical to the right side, the corresponding position on the left was marked and defined as NMTrP. Figures 1(a) and 1(b) show the distribution of MTrP and NMTrP. A graphical diagram of the experimental setup is shown in Figure 1(c). All participants were asked to relax during the induction of stimulating pain using 25 Newton pressure from the force gauge [22, 23] on MTrPs and NMTrPs, respectively.

An initial resting state session of 8 min was measured using fNIRS for each subject for the whole experiment. During this session, the participants were required to remain as motionless as possible with their eyes closed and minds relaxed. Secondly, pain stimulation was induced by a force gauge on the position of NMTrP for 8 min. This session was defined as an NMTrP state, and fNIRS signals were collected simultaneously. The subjects were then asked to stop (but not sleep) and take off the fNIRS head cap for 2 h, helping subjects restore to their resting state. Lastly, pain stimulation was induced on the position of the latent MTrP for 8 min.

### 2.3. Data Acquisition

**Figure 2:**
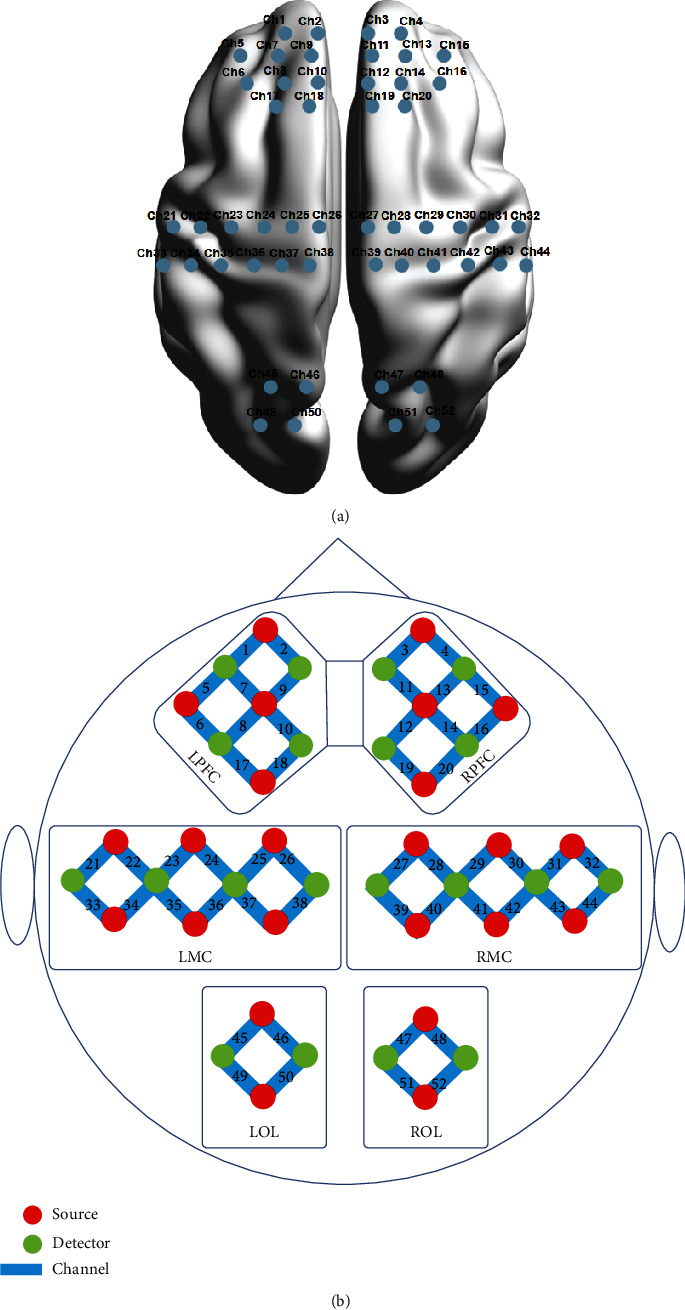
(a) Superior view 3D topography of optode positions in PFC-, MC-, and OL-related regions. (b) Configuration of 52 channels corresponding to the 10/10 electrode positions. Ch: channel; L: left; R: right; PFC: prefrontal cortex; MC: motor cortex; OL: occipital lobe.

In this study, a continuous-wave fNIRS system (Nirsmart, Danyang Huichuang Medical Equipment Co., Ltd., China) was utilized, using light at 760 and 850 nm wavelengths to measure the changes in the concentrations of oxygenated hemoglobin (*Δ*[oxy-Hb]) with a sampling rate of 10 Hz. A total of 52 channels, set up as 24 source optodes and 16 detector optodes, were symmetrically positioned over the L/R PFC, L/R MC, and L/R OL regions. The channel configuration and regions of interest areas are illustrated in Figure 2. Using the calibration function of the instrument and the corresponding template, the channels were determined to precisely fill the corresponding of the 10/10 electrode positions with different head sizes of the participants [24].

### 2.4. Data Preprocessing and Analysis

Firstly, we removed the channel with invalid horizontal line signals of the collected fNIRS signals by visual inspection for each subject. Then, the fNIRS signals were filtered by a Butterworth filter with a cutoff frequency 0.005-2 Hz. The motion artifacts in fNIRS signals were detected and removed by moving the standard deviation and spline interpolation [25]. Then, the fluctuation signals of *Δ*[oxy-Hb] were deduced using the modified Beer-Lambert law [26]. Wavelet transform was adopted to obtain the phase dynamic information of the oscillation signals [27]. The phase dynamic information identified the effective network model among the 52 channels of the fNIRS measurement, which was established based on the coupling function [28]. The parameters describing the coupling model were inferred by dynamic Bayesian inference, which reveals the functional rules of interaction in the brain dynamical system [29]. The coupling relationships (including coupling strength and direction) between every two channels were described quantitatively by the model parameters. The instantaneous phases and their possible relationships with wavelet phase coherence were identified.

Collected fNIRS signals can be divided into evoked/nonevoked neurovascular coupling and systematic physiological interference. Therefore, mechanisms such as endothelial-derived nitric oxide, vascular myogenic response, and sympathetic nervous system could overlap and affect wavelet signals. The wavelet amplitude (WA), coupling strength (CS), and main coupling direction (mCD) were calculated in five intervals [30, 31]: I: cardiac activity (0.6–2 Hz), II: respiratory activity (0.145–0.6 Hz), III: myogenic activity (0.052–0.145 Hz), IV: neurogenic activity (0.021–0.052 Hz), and V: endothelial cell metabolism (0.0095-0.021 Hz), to describe the frequency-specific cortical activities and EC network, and intervals III, IV, and V were exhibited to reveal different relationships [32, 33].

### 2.5. Statistical Analysis

The Kolmogorov-Smirnov and Levene tests were applied to test the data's variance, normality, and homogeneity at the group level. Statistical analyses for the wavelet amplitude (WA), effective connectivity (EC), and main coupling direction (mCD) were evaluated using a one-way ANOVA in IBM SPSS (V 26.0). An *α* level of 0.05 and 95% confidence intervals were assumed to be statistically significant for all analyses, except comparison of EC among three states in three different intervals where the *α* value was adjusted as 0.0167 (0.05/3).

## 3. Results

### 3.1. Basic Parameters' Changes

The latent MTrP characteristics of the subjects are presented in Table 1. There was no statistical difference in BP and HR, but the participants' pain degree changed apparently in VAS results before and during stimuli of the latent MTrP.

### 3.2. Wavelet Amplitude Analysis

Wavelet amplitude (WA) reflects the fluctuation magnitude of the original signal in a specific frequency, so it serves as an index of power that describes the activity intensity of the cortical region [34]. The following graphs depict the changes in WA for the III, IV, and V intervals.

**Figure 3:**
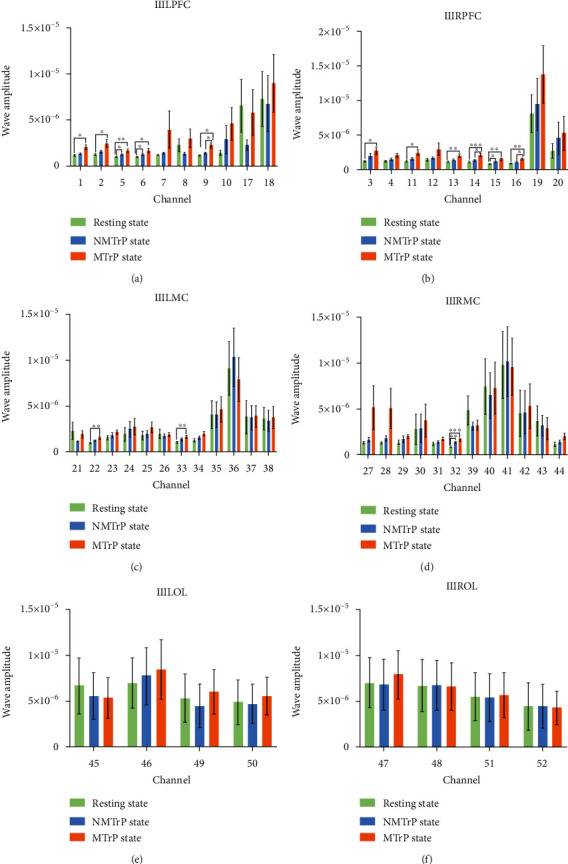
Comparison of wavelet amplitude (WA) changes among three stages in the frequency interval III: PFC (a and b), MC (c and d), and OL (e and f). Error bars are mean ± s.e.m.∗: *p* < 0.05; ∗∗: *p* < 0.01; ∗∗∗: *p* < 0.001. MTrP: myofascial trigger point; NMTrP: nonmyofascial trigger point; L: left; R: right; PFC: prefrontal cortex; MC: motor cortex; OL: occipital lobe.

Figure 3 shows the WA in interval III (myogenic activity (0.052–0.145 Hz)). Within LPFC, there were five channels (1, 2, 5, 6, and 9) with statistical differences between resting state and MTrP state, two channels (5 and 6) between resting state and NMTrP state, and only one channel (9) between the NMTrP state and MTrP state. Within RPFC, significant differences have been found in six channels (3, 11, 13, 14, 15, and 16) between the resting state and MTrP state, only one channel (15) between the resting state and NMTrP state, and two channels (14 and 16) between NMTrP state and MTrP state. Within LMC, two channels (22 and 33) showed statistical differences between the resting state and MTrP state; no statistical differences were found between the resting state and NMTrP state or NMTrP state and MTrP state. Within RMC, only one channel (32) showed a significant difference between the resting state and MTrP state; only one channel (32) illustrated a statistically significant difference between resting state and NMTrP state and no statistical differences between NMTrP state and MTrP state. Within LOL and ROL, no statistical differences were ascertained.

**Figure 4:**
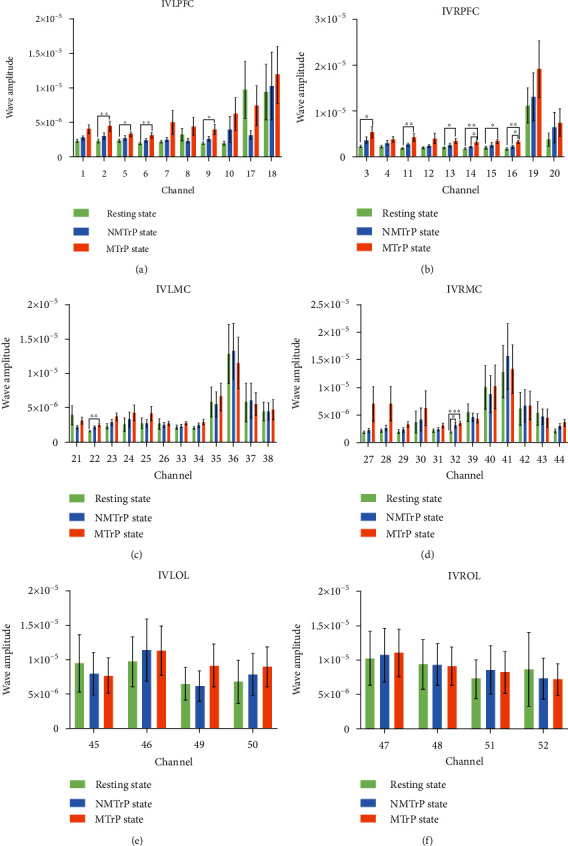
Comparison of wavelet amplitude (WA) changes among three stages in the frequency interval IV: PFC (a and b), MC (c and d), and OL (e and f). Error bars are mean ± s.e.m. ∗: *p* < 0.05; ∗∗: *p* < 0.01; ∗∗∗: *p* < 0.001. MTrP: myofascial trigger point; NMTrP: nonmyofascial trigger point; L: left; R: right; PFC: prefrontal cortex; MC: motor cortex; OL: occipital lobe.

Figure 4 illustrates data for WA in the interval IV (neurogenic activity (0.021–0.052 Hz)). Within LPFC, four channels (2, 5, 6, and 9) yielded statistical differences between the resting state and MTrP state; no statistical differences were observed for resting state vs. NMTrP state and NMTrP state vs. MTrP state. Within RPFC, six channels (3, 11, 13, 14, 15, and 16) yielded significant differences between resting state and MTrP state. When comparing the resting state with NMTrP state, no channel showed significant differences, while two channels (14 and16) showed significant differences between the NMTrP state and MTrP state. Within LMC, only one channel (22) exhibited a significant difference between the resting state and MTrP state. No statistical difference was found comparing the resting state vs. NMTrP state, or NMTrP state vs. MTrP state. Within RMC, only one channel (32) exhibited significant differences between the resting state and MTrP state. Only one channel (32) yielded significant differences between the resting state vs. NMTrP state, and no channel recorded a statistical difference between the NMTrP state and MTrP state. Within LOL and ROL, there were no statistical differences detected.

**Figure 5:**
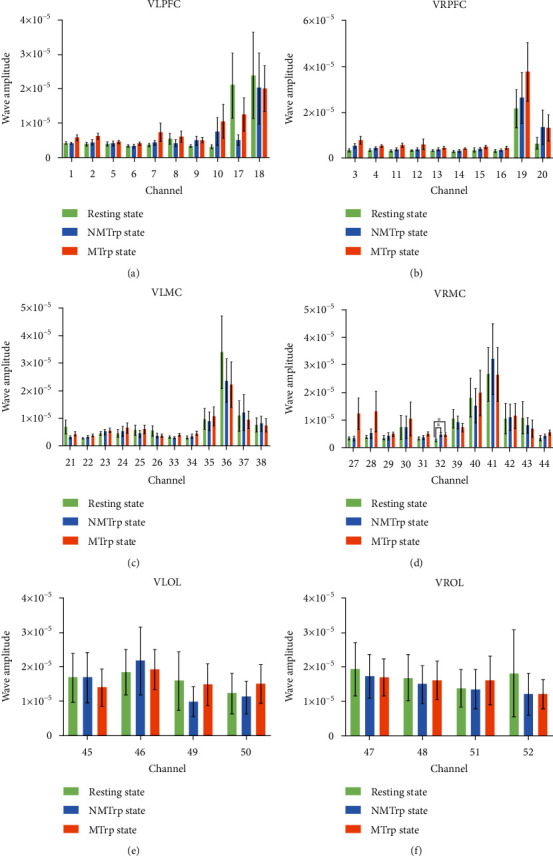
Comparison of wavelet amplitude (WA) changes among three stages in frequency interval V: PFC (a and b), MC (c and d), and OL (e and f). Error bars are mean ± s.e.m.∗: *p* < 0.05; ∗∗: *p* < 0.01; ∗∗∗: *p* < 0.001. MTrP: myofascial trigger point; NMTrP: nonmyofascial trigger point; L: left; R: right; PFC: prefrontal cortex; MC: motor cortex; OL: occipital lobe.

Figure 5 shows the WA in interval V (endothelial cell metabolism (0.0095-0.021 Hz)). Within LPFC, no existing statistical differences were recorded. Within RPFC, no channel recorded a statistical difference between the resting state vs. MTrP state, resting state vs. NMTrP state, and NMTrP state vs. MTrP state. Within LMC, no channel yielded a statistical difference when comparing the resting state with the MTrP state, resting state with the NMTrP state, and the NMTrP state with MTrP state. Within RMC, only one channel (32) significantly differed between the resting state and MTrP state. One channel (32) was significantly different between the resting state and NMTrP state. No channel was statistically different between the NMTrP and MTrP states. Within LOL and ROL, no statistical differences were found.

The results demonstrated that WA increased in the LPFC, RPFC, LMC, and RMC channels in intervals III and IV and only one RMC channel in the interval V (Figures 3–5) in the MTrP state compared to that in the resting state. In the NMTrP state, the WA in the RMC channel of interval III (Figure 3) was significantly higher than that in the resting state. Although an increasing trend was observed between the MTrP state and NMTrP state, it was not statistically significant. Thus, there seems to be an increasing trend of WA values in the MTrP and NMTrP states, compared with the resting state in the intervals III, IV, and V.

### 3.3. Effective Connectivity Analysis

To further investigate the neurovascular coupling interaction among these cortexes, the effective connectivity (EC), which refers explicitly to the influence that one neural system exerted over another to help describe the causality of interactions among brain regions, was adopted [35]. EC is assessed by coupling strengths.

**Figure 6:**
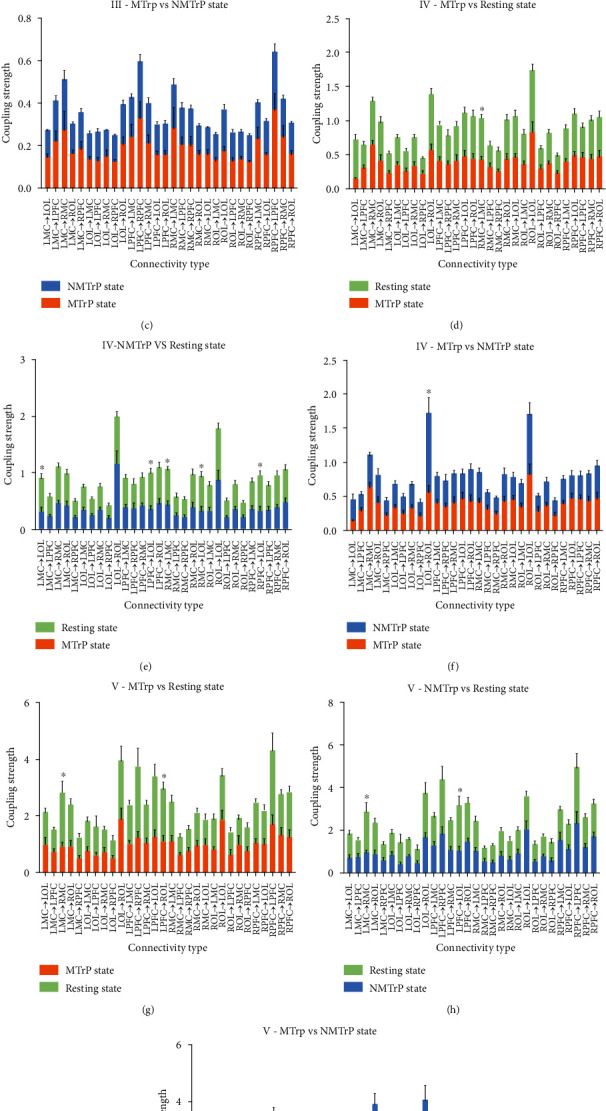
Comparison of region-wise EC among three states in three different intervals: interval III (a–c), interval IV (d–f), and interval V (g–i). Error bars are mean ± s.e.m. No significant difference (*p* > 0.0167); ∗: *p* < 0.0167. MTrP: myofascial trigger point; NMTrP: nonmyofascial trigger point; L: left; R: right; PFC: prefrontal cortex; MC: motor cortex; OL: occipital lobe.

Figure 6 shows the significant changes in EC values between two states in three different intervals, individually. No significant differences were recorded in interval III (Figures 6(a)–6(c)). In interval IV (Figures 6(d)–6(f)), comparing the MTrP and resting states, the connectivity of RMC→LMC showed a statistical difference. Considering NMTrP state vs. resting state, five significant differences in LPFC→LOL, RPFC→LOL, LMC→LOL, RMC→LMC, and RMC→LOL were found. Only the connectivity of LOL→ROL is statistically different when comparing the NMTrP and MTrP states. In the frequency interval V (Figures 6(g)–6(i)), significant differences were detected between the MTrP state and the resting state in the two connections (LPFC→ROL and LMC→RMC). Considering NMTrP state vs. resting state also yielded statistical differences in the two conditions (LPFC→LOL and LMC→RMC). A decreasing trend of EC values in the MTrP and NMTrP states was apparent, compared with the resting state in the intervals III, IV, and V.

### 3.4. Main Coupling Direction Analysis

**Figure 7:**
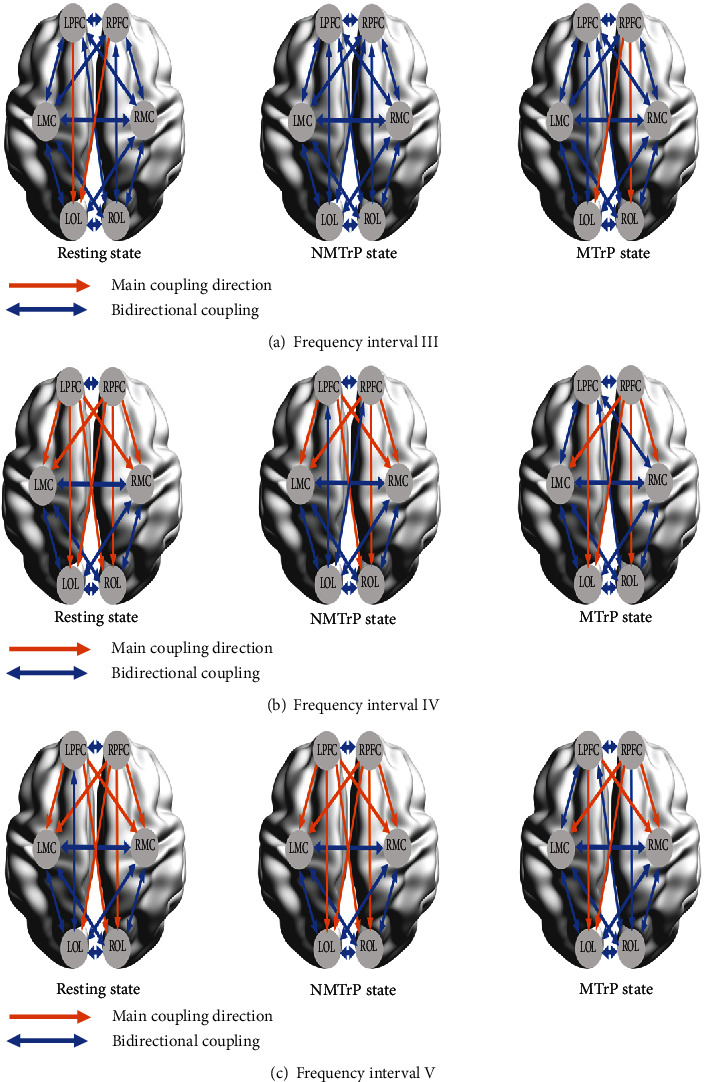
Illustration of frequency-specific interregional main coupling directions (mCD) (resting state, NMTrP state, and MTrP state) among the six brain regions in frequency intervals III (a), IV (b), and V (c), respectively. MTrP: myofascial trigger point; NMTrP: nonmyofascial trigger point; L: left; R: right; PFC: prefrontal cortex; MC: motor cortex; OL: occipital lobe.

Main coupling direction (mCD) calculations were undertaken to investigate how every significant interaction of all possible pair channels between 2 brain regions can exhibit different dominant functions. When the oscillator value of CS_*i*→*j*_ exceeded CS_*j*→*i*_, it would be defined *i*→*j* as the main coupling direction (mCD) of the interaction between channel *i* and channel *j*, for the coupling parameters of each channel pair. Significant differences can demonstrate interregional mCD, suggesting that a predominant coupling function between the two regions is possible. Otherwise, it would be considered bidirectional coupling [36]. The frequency-specific interregional coupling directions among the six brain regions in the resting state, NMTrP state, and MTrP state, respectively, are displayed below (in Tables 2 and 3 and Figure 7).

In interval III, in the resting state, two mCDs were detected, LPFC→LOL (*p* = 0.009) and RPFC→LOL (*p* = 0.004). In the MTrP state, two mCDs were detected, including RPFC→LOL (*p* = 0.03) and RPFC→ROL (*p* = 0.037). These mCDs are illustrated in Figure 7(a).

In interval IV, resting state yielded four mCDs dominant in LPFC and RPFC and no mCD from either MC or OL. In the NMTrP state, there were three mCDs from LPFC, three mCDs from RPFC, and no mCD from either MC or OL. In the MTrP state, only one mCDs from LPFC, four mCDs from RPFC, and no mCD from either MC or OL were found (Table 2 and Figure 7(b)).

In interval V, in the resting state, three mCDs were dominant in LPFC, four mCDs in RPFC, and no mCD from either MC or OL. In the NMTrP state, there were four mCDs from LPFC, four mCDs from RPFC, and no mCD from either MC or OL. In the MTrP state, two mCDs from LPFC, three mCDs from RPFC, and no mCD from either MC or OL were detected (Table 3 and Figure 7(c)).

In the NMTrP state, the interregional mCD was primarily observed in intervals IV and V from LPFC to LMC, RMC, and ROL; from RPFC to LMC, RMC, and ROL; and from LPFC to LOL and RPFC to LOL in the interval V. In the MTrP state, the interregional mCD was primarily found in intervals IV and V from RPFC to LMC and RMC, LPFC, to LOL, in the intervals III, IV, and V from RPFC to LOL, in intervals III and IV from RPFC to ROL, and in interval V from LPFC to RMC. Thus, the interregional mCD tends to be from LPFC and RPFC in intervals III, IV, and V.

## 4. Discussion

This study investigated the nonsensory cortical reactions, including changes in cortex neuronal activity and brain effective connectivity induced by a latent MTrP with fNIRS. WA has been used to describe cortical activity intensity, and EC refers to the interactional directional connectivity among regions. We observed an increasing trend of WA and a decreasing trend of EC values in the MTrP and NMTrP states, compared with the resting state in intervals III, IV, and V. Notably, the interregional mCD preferred to be from LPFC and RPFC in intervals III, IV, and V.

Previous studies have found that pain activates a wide range of cortical and subcortical areas, not only from one region. Except for the primary and secondary somatosensory areas, it includes the motor-related cortex, anterior cingulate cortex, and prefrontal cortex [37]. Moreover, this study further found that the nonsensory cortex including PFC, MC, and OL was activated when latent MTrPs were stimulated in MPS patients, and during which processes, complex mechanisms can be activated [30, 38, 39].

The present study shows that the MC can be activated either for the MTrP or NMTrP state, but the PFC has been activated only for the MTrP state (Figures 3–5). Previous studies also show that the PFC experiences an abnormal increase in activity during chronic pain [40, 41], contributing to the PFC's role in the transition between resting and task-processing states [29]. All these findings indicate that activation of the MC occurs within the cerebral cortex when various pain occurs, but PFC activation occurs specifically in MTrP-related changes. In contrast to the NMTrP state, some channels of LPFC and RPFC were significantly activated in the intervals III and IV in the MTrP state. Such activation may be related to the increased sensitivity, decrease of the pain threshold, and possibly the increase of the receptive field of the MTrP state correlated with peripheral and central sensitization [32, 42].

In addition, the connectivity between coupling cortexes also varied (Figure 6). The term connectivity can refer to different and interrelated aspects of brain organization. A fundamental distinction is between structural, functional, and effective connectivity. Among them, functional and effective connectivity refers to the functional connection between brain regions, based on a particular cognitive process [43]. The EC is a directional connectivity that depends on interactions among regions. In this study, its value in the connectivity of LOL→ROL in the MTrP state decreased in interval IV compared with the NMTrP state, suggesting that OL may play a role in the regulation of MTrP and may relate to specialized cells or groups of cells existing in the occipital cortex that can recognize the spatial location of pain [44]. Notably, the interregional mCD (Figure 7) was primarily shifted from unidirectional to bidirectional connection in responding to the MTrP state compared with the NMTrP state [36, 45]. These changes suggest that the PFC regulation on other brain regions was weakened, while the feedback regulation of other brain regions on the PFC was enhanced during the MTrP state rather than in the NMTrP state.

Previous studies also found that the PFC is an essential node in brain connectivity, which can play a higher cognitive role in pain processing, and regulates pain awareness and pain response through the redistribution of attention [46, 47]. The PFC was not only related to other parts of the frontal lobe but also to other brain areas through such structures as the frontooccipital tracts [48, 49], which may be the basis of PFC regulation of network connections in other brain areas.

Further evidence further suggests that chronic pain is associated with structural and functional changes in M1 and affects the motor reflex arc (inputs and outputs) [50, 51]. Consequently, these changes may correlate with the increased regulation of the prefrontal cortex by motor areas that plan the avoidance response to pain. In a recent report, it has been demonstrated that long-term noxious stimulation can lead to changes in brain structure and function and dysfunction of neurovascular coupling [52]. It can also provide a possible explanation for cortical changes related to the latent MTrP.

## 5. Limitations

Firstly, the sample size is relatively small and needs to be expanded in future studies. Secondly, longitudinal experiments are needed to characterize further cortical changes induced by the latent and active MTrPs.

## 6. Conclusions

This study suggests that the nonsensory cortex, including the PFC, MC, and OL, can be involved in the reaction after stimulation of the latent MTrP. Additionally, high activation of the PFC and its weakened regulation in other brain regions were also characterized. The PFC shows high relevance and responsibility for regulating latent MTrP. However, further studies are needed to understand the MPS clinical outcomes regarding the findings of this study.

## Figures and Tables

**Table 1:** Fundamental information of experimental subjects in the latent MTrP before and during stimuli.

Parameter	VAS	BP (mmHg)		HR (times/min)
		SBP	DBP	
Before stimuli	0	119.00 ± 15.95	70.88 ± 8.13	73.13 ± 7.95
During stimuli	7.53 ± 0.72^∗∗∗^	120.00 ± 16.46	71.50 ± 8.16	72.38 ± 9.12

VAS: visual analogue scale; BP: blood pressure; SBP: systolic blood pressure; DBP: diastolic blood pressure: HR: heart rate; MTrP: myofascial trigger point; min: minute. ∗∗∗: *p* ≤ 0.001. Error bars are mean ± s.e.m.

**Table 2:** Statistical results of mCDs among 6 brain regions in Interval IV.

mCD	Resting state	NMTrP state	MTrP state
LPFC→RPFC	p = 0.869	p = 0.732	p = 0.165
LPFC→LMC	p = 0.020^∗^	p = 0.013^∗^	p = 0.090
LPFC→RMC	p = 0.035^∗^	p = 0.004^∗∗^	p = 0.297
LPFC→LOL	p ≤ 0.001^∗∗∗^	p = 0.135	p = 0.021^∗^
LPFC→ROL	p = 0.002^∗∗^	p = 0.005^∗∗^	p = 0.174
RPFC→LMC	p = 0.003^∗∗^	p = 0.022^∗^	p = 0.005^∗∗^
RPFC→RMC	p = 0.001^∗∗^	p =0.002^∗∗^	p =0.016^∗^
RPFC→LOL	p ≤ 0.001^∗∗∗^	p = 0.079	p = 0.015^∗^
RPFC→ROL	p = 0.001^∗∗^	p = 0.003^∗∗^	p = 0.016^∗^
LMC→RMC	p = 0.788	p = 0.675	p = 0.823
LMC→LOL	p = 0.052	p = 0.805	p = 0.138
LMC→ROL	p = 0.227	p = 0.429	p = 0.563
RMC→LOL	p = 0.05	p = 0.883	p = 0.146
RMC→ROL	p = 0.201	p = 0.657	p = 0.486
LOL→ROL	p = 0.526	p = 0.307	p = 0.133

mCD: main coupling direction; L: left; R: right; PFC: prefrontal cortex; MC: motor cortex; OL: occipital lobe. *p* > 0.05, no statistic difference existed; ∗: significant difference; ∗: *p* < 0.05; ∗∗: *p* < 0.01; ∗∗∗: *p* ≤ 0.001.

**Table 3:** Statistical results of mCDs among six brain regions in Interval V.

mCD	Resting state	NMTrP state	MTrP state
LPFC→RPFC	p = 0.951	p = 0.394	p = 0.205
LPFC→LMC	p = 0.005^∗∗^	p = 0.013^∗^	p = 0.095
LPFC→RMC	p ≤ 0.001^∗∗∗^	p = 0.002^∗∗^	p = 0.035^∗^
LPFC→LOL	p = 0.052	p = 0.002^∗∗^	p = 0.023^∗^
LPFC→ROL	p ≤ 0.001^∗∗∗^	p ≤ 0.001^∗∗∗^	p = 0.057
RPFC→LMC	p = 0.001^∗∗^	p = 0.022^∗^	p ≤ 0.001^∗∗∗^
RPFC→RMC	p = 0.002^∗∗^	p ≤ 0.001^∗∗∗^	p = 0.039^∗^
RPFC→LOL	p = 0.040^∗^	p = 0.004^∗∗^	p = 0.047^∗^
RPFC→ROL	p = 0.007^∗∗^	p ≤ 0.001^∗∗∗^	p = 0.083
LMC→RMC	p = 0.264	p = 0.471	p = 0.432
LMC→LOL	p = 0.684	p = 0.532	p = 0.374
LMC→ROL	p = 0.100	p = 0.835	p = 0.674
RMC→LOL	p = 0.599	p = 0.284	p = 0.344
RMC→ROL	p = 0.191	p = 0.983	p = 0.942
LOL→ROL	p = 0.382	p = 0.428	p = 0.949

mCD: main coupling direction; L: left; R: right; PFC: prefrontal cortex; MC: motor cortex; OL: occipital lobe. *p* > 0.05, no statistic difference existed; ∗: significant difference. ∗: *p* < 0.05; ∗∗: *p* < 0.01; ∗∗∗: *p* ≤ 0.001.

## Data Availability

Data is available on request from the corresponding author (e-mail: yonghuiw6606@126.com).
